# Circuit mechanisms and computational models of REM sleep

**DOI:** 10.1016/j.neures.2018.08.003

**Published:** 2019-03

**Authors:** Charlotte Héricé, Amisha A. Patel, Shuzo Sakata

**Affiliations:** Strathclyde Institute of Pharmacy and Biomedical Sciences, University of Strathclyde, 161 Cathedral Street, Glasgow G4 0RE, UK

**Keywords:** REM sleep, Brainstem, Hypothalamus, Computational model, Cell type, Brain state

## Abstract

Rapid eye movement (REM) sleep or paradoxical sleep is an elusive behavioral state. Since its discovery in the 1950s, our knowledge of the neuroanatomy, neurotransmitters and neuropeptides underlying REM sleep regulation has continually evolved in parallel with the development of novel technologies. Although the pons was initially discovered to be responsible for REM sleep, it has since been revealed that many components in the hypothalamus, midbrain, pons, and medulla also contribute to REM sleep. In this review, we first provide an up-to-date overview of REM sleep-regulating circuits in the brainstem and hypothalamus by summarizing experimental evidence from neuroanatomical, neurophysiological and gain- and loss-of-function studies. Second, because quantitative approaches are essential for understanding the complexity of REM sleep-regulating circuits and because mathematical models have provided valuable insights into the dynamics underlying REM sleep genesis and maintenance, we summarize computational studies of the sleep-wake cycle, with an emphasis on REM sleep regulation. Finally, we discuss outstanding issues for future studies.

## Highlights

• REM sleep was discovered in the 1950s.
• Many hypothalamic and brainstem areas have been found to contribute to REM sleep.
• An up-to-date picture of REM-sleep-regulating circuits is reviewed.
• A brief overview of computational models for REM sleep regulation is provided.
• Outstanding issues for future studies are discussed.

## 1 Introduction

Brain states vary from moment to moment throughout the day. Humans typically cycle between three major behavioral states: wakefulness, rapid eye movement (REM) sleep and non-REM (NREM) sleep, with additional stages of NREM sleep. REM and NREM sleep have distinct characteristics. For example, NREM sleep is characterized by slow, large-amplitude fluctuations of cortical electroencephalograms (EEGs) whereas REM sleep is characterized by fast, small-amplitude fluctuations of EEGs. Although these sleep states are closely related with each other with respect to neural mechanisms and functions (Brown et al., 2012; Hobson and Pace-Schott, 2002; Pace-Schott and Hobson, 2002; Scammell et al., 2017; Stickgold et al., 2001; Weber and Dan, 2016), this review article particularly focuses on the mechanisms underlying REM sleep.

REM sleep is associated with vivid dreaming, rapid eye movement, muscle atonia and other body homeostatic signatures. Electrophysiological characteristics of REM sleep include desynchronized cortical EEG, hippocampal theta waves and ponto-geniculo-occipital (PGO) waves (Aserinsky and Kleitman, 1953; Brown et al., 2012; Dement and Kleitman, 1957; Jouvet, 1962; Luppi et al., 2012; Peever and Fuller, 2017; Scammell et al., 2017; Weber and Dan, 2016; Callaway et al., 1987; Datta, 1997). REM sleep is also known as “paradoxical sleep” because the desynchronized EEG observed during REM sleep resembles that during wakefulness, but without muscle tone (Jouvet et al., 1959). REM sleep also contains phasic and tonic periods: phasic periods are characterized by bursts of rapid eye movements whereas no rapid eye movements occur during tonic periods (Wehrle et al., 2007; Moruzzi, 1963). Although detailed characteristics (e.g., REM sleep duration, eye movements) vary across species, birds and lizards also exhibit similar electrophysiological features of REM sleep, suggesting that REM sleep evolved in a common ancestor early in amniote evolution (Low et al., 2008; Monnier, 1980; Siegel, 1995; Joiner, 2016; Shein-Idelson et al., 2016).

The sleep stage with REM and desynchronized EEG activity was originally discovered in humans in the 1950s (Aserinsky and Kleitman, 1953; Dement and Kleitman, 1957), and subsequently confirmed in cats (Dement, 1958). Jouvet et al. comprehensively described the main characteristics of REM sleep and established the notion that the pons is responsible for REM sleep (Jouvet, 1962; Jouvet and Michel, 1959).

Since these landmark studies, with the advent of technological advancements, our understanding of the neurobiology underlying REM sleep regulation has expanded considerably. In the late 1950s and 60s, lesion, electrophysiological and pharmacological experiments identified the brainstem structures and neurotransmitters responsible for REM sleep (Jouvet, 1962). In the 70s, unit recording experiments identified brainstem neurons which are exclusively active (REM-on) or silent (REM-off) during REM sleep in cats (McCarley and Hobson, 1971; Hobson et al., 1975; Jouvet, 1972). Subsequently, as novel approaches ranging from Fos mapping and juxtacellular recording to recent genetic and molecular technologies have been adopted, various hypothalamic and brainstem nuclei have been identified to contribute to REM sleep (Boissard et al., 2002; Hassani et al., 2009; Krenzer et al., 2011; Lu et al., 2006b; Luppi et al., 2017; Scammell et al., 2017; Weber et al., 2015; Weber and Dan, 2016; Brown et al., 2012).

**Fig. 1:**
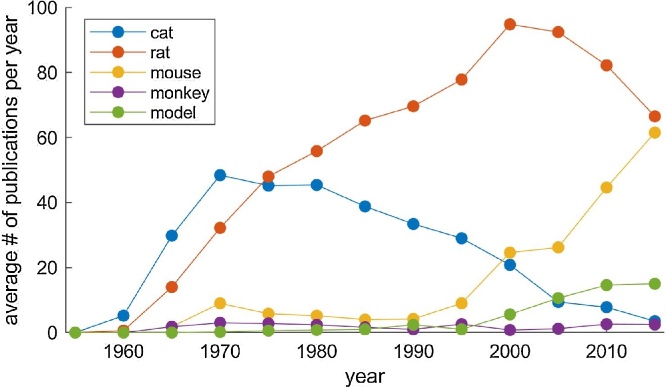
**Publication records on REM sleep research**. The number of publication records on REM sleep was extracted from the PubMed database for each animal species. The publication records of computational studies on sleep-wake cycle were also extracted (‘model’).

Trends in choice of animal models have also changed (Fig. 1): Although the cat model initially dominated the field, the use of rats had become increasingly popular until the 2000s. This is probably due to their smaller size and the development of anatomical, histochemical and electrophysiological methods. Over the past decade, the use of mice has rapidly gained momentum due to the revolution of molecular genetic approaches for systems-level studies, such as viral tracing, optogenetics and chemogenetics. While there is no doubt that mice will soon become a dominant species in this field, it should be also noted that REM sleep has been confirmed in different species including the *Pogona* dragons (Shein-Idelson et al., 2016), indicating the importance of comparative studies.

In addition to animal studies, computational/mathematical studies of sleep-wake cycles have also made important contributions to this field since the pioneering studies by McCarley and Hobson (1975) and Borbely (1982). Given the complexity of sleep regulatory circuits, such quantitative approaches will become increasingly essential. Indeed, there has been an upward trend of publication records over the past two decades (Fig. 1).

In the present review, we summarize the current status of our understanding of REM sleep regulation, with a focus on the circuit mechanisms and computational models. First, we summarize the current understanding of key brain regions and neuropeptides within REM sleep-regulating circuits. Second, we cover a range of computational models to explain the sleep-wake cycle. Finally, we discuss outstanding issues and future challenges in this field. Readers should also refer to other review articles (Brown et al., 2012; Luppi et al., 2013a; Peever and Fuller, 2017; Saper et al., 2010; Scammell et al., 2017; Weber and Dan, 2016).

## 2 REM sleep-regulating circuits

**Fig. 2:**
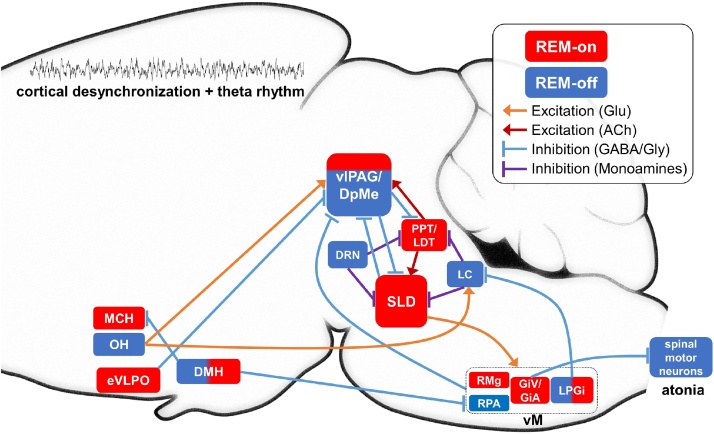
**Diagram of REM sleep-regulating circuits.** Brainstem and hypothalamic areas described in the main text are shown, with a simplified view of activity during REM sleep as well as connectivity. DMH, dorsomedial hypothalamus; DpMe, dorsal part of the deep mesencephalic reticular nuclei; DRN, dorsal raphe nucleus; eVLPO, extended area of the ventrolateral preoptic area; GiA, alpha gigantocellular nucleus; GiV, ventral gigantocellular nucleus; LC, locus coeruleus; LDT, laterodorsal tegmental nucleus; LPGi, lateral paragigantocellular nucleus; MCH, melanin concentrating hormone neurons; OH, orexin/hypocretin neurons; PPT, pedunculopontine tegmental nucleus; RMg, raphe magnus; RPA, nucleus raphe pallidus; SLD, sublaterodorsal nucleus; vlPAG, ventrolateral periaqueductal gray. Glu, glutamate; ACh, acetylcholine; GABA, γ-aminobutyric acid; Gly, glycine.

REM sleep-regulating circuits are widespread throughout the brainstem (midbrain, pons, and medulla) and the hypothalamus and involve a range of neurotransmitters and neuropeptides. In this section, we survey the literature on key components of REM sleep-regulating circuits within the brainstem and hypothalamus (Fig. 2).

### 2.1 Brainstem REM sleep-regulating circuits

Research on brainstem REM sleep-regulating circuits has a long history since pioneering studies by Jouvet and his colleagues (Jouvet and Michel, 1959; Jouvet, 1962). Despite numerous efforts over the past six decades, a comprehensive picture of brainstem REM sleep-regulating circuits is still lacking. Here we focus on the key brainstem nuclei involved in REM sleep regulation, by summarizing their 1) anatomical features, 2) neural activity during REM sleep and 3) gain- and loss-of-function studies.

#### 2.1.1 Sublaterodorsal nucleus

The sublaterodorsal (tegmental) nucleus (SLD) in rodents has long been implicated in REM sleep genesis and muscle atonia (Luppi et al., 2006, 2012; Luppi et al., 2017; Scammell et al., 2017).

***Anatomy.*** The SLD is located immediately ventral from the periaqueductal gray (PAG) and corresponds to the rostral part of the subcoeruleus nucleus. It is equivalent to the peri-locus coeruleus alpha (peri-LCα) in the cat (Sakai et al., 1979, 2001) and is composed of glutamatergic, GABAergic and cholinergic neurons (Sakai et al., 2001; Boissard et al., 2002).

The SLD receives glutamatergic inputs from the lateral and ventrolateral PAG (vlPAG), the primary motor cortex, the bed nucleus of the stria terminalis (BNST) and the central nucleus of the amygdala. It also receives GABAergic inputs from the mesencephalic, pontine reticular nuclei and to a lesser extent the parvicellular reticular nucleus and contralateral SLD (Boissard et al., 2003; Lu et al., 2006b). Although a recent study with a cell-type-specific tracing approach confirmed the projection from vlPAG GABAergic neurons onto glutamatergic neurons in the SLD (Weber et al., 2018), input specificity onto each SLD cell-type remains to be fully characterized.

SLD glutamatergic neurons project rostrally to the intralaminar nuclei of the thalamus, posterior hypothalamus, and basal forebrain (BF), and caudally to glycinergic neurons in the raphe magnus (RMg), ventral and alpha gigantocellular nuclei (GiA and GiV) and the lateral paragigantocellular nucleus (LPGi). These rostral and caudal projections are thought to be responsible for cortical desynchronization and muscle atonia, respectively (Sakai et al., 1979; Jones, 1991a; Boissard et al., 2002).

***Activity.*** SLD glutamatergic neurons are primarily REM-on, meaning that they are more active during REM sleep compared to non-REM (NREM) sleep and wakefulness (Sakai, 1985; Sakai and Koyama, 1996; Lu et al., 2006b; Clement et al., 2011). It has been repeatedly shown that SLD glutamatergic neurons strongly express Fos after prolonged REM sleep in rats and mice (Clement et al., 2011; Krenzer et al., 2011). Although SLD GABAergic neurons have been initially reported to be REM-on (Lu et al., 2006b), a subsequent report does not support this observation (Sapin et al., 2009). SLD cholinergic neurons do not appear to be REM-on based on Fos mapping (Verret et al., 2005) although an extracellular recording study demonstrated that a subpopulation of SLD cholinergic neurons are active during both REM sleep and wakefulness (Sakai, 2012). Thus, although the SLD has been implicated in REM sleep genesis, future studies need to reconcile the heterogeneity of state-dependent and cell-type-specific firing within the SLD.

***Function.*** Pharmacologically GABAa antagonists (bicuculine or gabazine) and glutamate agonist can induce REM sleep (Boissard et al., 2002; Xi et al., 1999; Onoe and Sakai, 1995), suggesting that tonic glutamatergic barrage and the removal of a tonic GABAergic tone can cause REM sleep. In contrast, cholinergic agonist (carbachol) induces REM when injected into the cat peri-LCα, but not in all cases when applied to the rat SLD (Boissard et al., 2002; Bourgin et al., 1995; Deurveilher et al., 1997; Gnadt and Pegram, 1986; Shiromani and Fishbein, 1986; Velazquez-Moctezuma et al., 1989). Given the contradictory results of cholinergic effects on REM sleep induction, further studies are necessary to quantitatively determine how the balance between different transmitter inputs onto SLD neurons can contribute to REM sleep induction.

Furthermore, a recent chemogenetic study demonstrated that glutamatergic neurons in the rostrolateral SLD (“Atoh1-E10.5-medial cells”) promote NREM sleep and inhibit REM sleep (Hayashi et al., 2015). These glutamatergic neurons project to GABAergic neurons in the deep mesencephalic reticular nucleus (DpMe), which negatively regulate REM sleep, possibly inhibiting REM sleep-promoting neurons in the SLD. Therefore, REM sleep-regulating circuitry within the SLD is more complex than previously appreciated.

The SLD also plays a causal role in muscle atonia. A focal lesion of the SLD or the deletion of glutamate signaling induces a REM sleep-like state, but without muscle atonia, thus implicating aberrant glutamatergic transmission in REM sleep behavioral disorder (Krenzer et al., 2011; Lu et al., 2006b). A prominent explanatory mechanism of muscle atonia is that because SLD glutamatergic neurons project to glycinergic/GABAergic neurons in the ventral medulla (vM), which in turn inhibit spinal motor neurons, the removal of SLD excitatory inputs onto vM cannot induce muscle atonia (Boissard et al., 2002; Luppi et al., 2017; Lu et al., 2006b). However, as we discuss below, distinct vM circuits have been implicated in REM sleep regulation and muscle atonia. Therefore, detailed circuit analysis between the SLD and vM is still required. Furthermore, the role of SLD GABAergic neurons is also unclear.

#### 2.1.2 Ventrolateral periaqueductal gray and deep mesencephalic reticular nucleus

The ventrolateral periaqueductal gray (vlPAG) together with the adjacent dorsal part of the deep mesencephalic reticular nucleus (DpMe) has been implicated in gating REM sleep by receiving inputs from the hypothalamus and other brainstem structures (Luppi et al., 2013a; Petitjean et al., 1975).

***Anatomy.*** The vlPAG is a part of the large midbrain structure, the PAG, and is located ventrolaterally within the caudal section of the PAG. vlPAG neurons are composed of glutamatergic, GABAergic and dopaminergic (DA) neurons. vlPAG DA neurons are often referred as to the dorso-caudal extension of the A10 group, which includes DA neurons in the dorsal raphe nucleus (DRN) (Cho et al., 2017; Dougalis et al., 2012; Hokfelt et al., 1984).

The vlPAG is an anatomical hub of REM sleep regulatory circuits within the brainstem. vlPAG neurons receive inputs from the forebrain, hypothalamus and brainstem, such as the central nucleus of amygdala, the zona incertia (ZI), the nucleus accumbens, the lateral hypothalamus, the lamina terminalis including the median preoptic nucleus (MnPO), the extended part of the ventrolateral preoptic nucleus (eVLPO), SLD, peduncluopontine tegmental nucleus/laterodorsal tegmental nucleus (PPT/LDT), and the vM (Boissard et al., 2003; Burgess et al., 2013; Clement et al., 2012; Hsieh et al., 2011; Liu et al., 2017; Uschakov et al., 2007, 2009; Weber et al., 2015; Zhang et al., 2013; Lu et al., 2006b).

Both GABAergic and non-GABAergic vlPAG neurons project to the SLD (Boissard et al., 2003; Weber et al., 2018) while GABAergic neurons also strongly innervate the DRN (Gervasoni et al., 2000) and the locus coeruleus (LC) (Weber et al., 2018). DA neurons have reciprocal connections with the medial prefrontal cortex, ventrolateral preoptic nucleus (VLPO), orexin/hypocretin neurons, LDT cholinergic neurons, and LC noradrenergic neurons (Lu et al., 2006a).

***Activity.*** REM-on and –off neurons are intermingled within the vlPAG and the DpMe, which reflects the complex nature of anatomical properties described above. While the majority of GABAergic neurons are REM-off, a subset of GABAergic neurons are also REM-on (Lu et al., 2006b; Sapin et al., 2009; Weber et al., 2018). REM-off neurons have been suggested to suppress SLD glutamatergic neurons during wakefulness and NREM sleep to prevent muscle atonia and REM sleep induction (Lu et al., 2006b).

***Function.*** The functional role of vlPAG neurons in REM sleep was first suggested with a lesion study carried out by Jouvet and his colleagues (Petitjean et al., 1975). While neurotoxic lesions in the vlPAG and DpMe promote REM sleep (Lu et al., 2006b), muscimol (GABAa agonist) application increases REM sleep in both cats (Sastre et al., 1996) and rats (Sapin et al., 2009). Furthermore, optogenetic activation of vlPAG GABAergic neurons suppresses REM sleep generation (Weber et al., 2015, 2018). As described above, GABAergic neurons in the DpMe receive inputs from glutamatergic neurons located in the rostrolateral SLD. Chemogenetic activation of DpMe GABAergic neurons inhibit REM sleep and promote NREM sleep, whereas chemogenetic silencing enhances REM sleep (Hayashi et al., 2015). These results are consistent with the notion that REM-off neurons located within the vlPAG and DpMe suppress REM sleep.

The function of REM-on neurons, however, remains elusive. Optogenetic stimulation of vlPAG GABAergic neurons can inhibit REM-on GABAergic neurons (Weber et al., 2018), suggesting intrinsic connections between REM-on and –off GABAergic neurons (Sapin et al., 2009; Weber et al., 2018). It has been proposed that REM-on GABAergic neurons in vlPAG can be activated by cholinergic inputs, resulting in the suppression of DRN serotonergic neurons (Yang and Brown, 2014). These results suggest that REM-on and REM-off GABAergic neurons in the vlPAG play antagonistic roles in REM sleep regulation.

vlPAG DA neurons are wake-promoting (Lu et al., 2006a), but the functional role of vlPAG glutamatergic neurons remains unclear. In addition, because the vlPAG receives inputs from the amygdala (Burgess et al., 2013) and has long been implicated in both fear-associated defensive behaviors and pain (Behbehani, 1995; Tovote et al., 2016), the relationship between anxiety/fear and sleep would be an interesting topic to explore.

#### 2.1.3 Ventral medulla

The functional role of the ventral medulla (vM) in REM sleep and muscle atonia has been actively debated (Luppi et al., 2012, 2006; Luppi et al., 2017; Sapin et al., 2009; Lu et al., 2006b; Weber et al., 2015). Accumulating evidence suggests that vM neurons play distinct roles in REM sleep and muscle atonia depending on circuits as summarized below.

***Anatomy.*** The vM is located within the ventrocaudal portion of the brainstem. It includes the raphe magnus (RMg), ventral and alpha gigantocellular nuclei (GiA and GiV) and the lateral paragigantocellular nucleus (LPGi), and other nuclei. GiA, GiV and RMg are located ventromedially within the vM, thus they are collectively known as the ventromedial medulla (vmM). Within the vM, diverse cell types are intermingled (Holmes and Jones, 1994; Leger et al., 2009; Rampon et al., 1996): while glutamatergic and GABAergic neurons are widely distributed, many GABAergic neurons are colocalized with glycine. Tyrosine hydroxylase positive neurons and a dense cluster of cholinergic neurons can be found in the LPGi and the nucleus ambiguus, respectively. Serotonergic neurons can be found in the RMg (B3 cell group) and the nucleus raphe pallidus (RPA) (B1 cell group).

The vM receives inputs from the spinal cord, medulla, pons, midbrain, hypothalamus, amygdala, and cortex (Sirieix et al., 2012; Van Bockstaele et al., 1989; Andrezik et al., 1981). Of these, GABA/glycinergic neurons receive strong glutamatergic inputs from the SLD (Fort et al., 2009; Luppi et al., 2013b).

GABA and/or glycine containing neurons in the vmM strongly innervate spinal motor neurons (Holstege, 1991; Holstege and Bongers, 1991). On the other hand, the LPGi project to the solitary tract, parabrachial nucleus, and Kӧlliker-Fuse nucleus (Guyenet and Young, 1987; Sirieix et al., 2012). LPGi GABAergic neurons provide inputs to the LC (Ennis and Aston-Jones, 1989; Sirieix et al., 2012). Interestingly, distinct populations of GABAergic neurons within the vM project rostrally to the vlPAG and caudally to the spinal cord (Weber et al., 2015) although the exact distribution of these populations within the vM remains unclear. Serotonergic cell groups (B1 and B3) in the vM also project to diverse areas, including the spinal cord, Kӧlliker-Fuse nucleus, LDT, SLD, LC, inferior colliculus, part of the thalamus, hypothalamus, BF and hippocampus (Loewy et al., 1981). Detailed cell type-specific projections, including glutamatergic, cholinergic, and aminergic projections, remain to be explored.

***Activity.*** GiV GABAergic neurons are REM-on in both cats (Siegel et al., 1979) and rodents (Maloney et al., 2000; Sapin et al., 2009). An enhanced Fos expression was observed following recovery from REM sleep deprivation in GiV, GiA and RMg GABAergic/glycinergic neurons, but to a lesser extent in the LPGi (Valencia Garcia et al., 2018). Indeed, LPGi GABAergic neurons show diverse firing responses including REM-on, REM-off, and other types (Sirieix et al., 2012). A recent optogenetic tagging study (Weber et al., 2015) showed that firing rates of vM GABAergic neurons increase gradually over ∼30 s before the NREM to REM transition and abruptly decrease at the end of REM sleep. Non-GABAergic neurons, on the other hand, respond with an increase in firing ∼10 s before the onset of REM sleep and a gradual decrease at the termination of REM sleep. Many of these non-GABAergic neurons were most active during running or moving. RPA serotonergic neurons are REM-off (Heym et al., 1982). Thus, the composition of REM-on and –off neurons vary across nuclei and cell types within the vM.

***Function.*** The functional role of vM neurons in REM sleep induction and muscle atonia has been actively debated (Fort et al., 2009; Luppi et al., 2013b; Valencia Garcia et al., 2018; Weber et al., 2015; Lu et al., 2006b). For example, GiV neurons have long been implicated in muscle atonia by receiving strong glutamatergic inputs from the SLD and co-releasing GABA and glycine onto spinal motor neurons (Luppi et al., 2012; Fort et al., 2009; Luppi et al., 2013b). However, lesioning the vM has had no effect on muscle atonia (Lu et al., 2006b). Weber and his colleague (Weber et al., 2015) recently demonstrated the causal role of the vM in REM sleep regulation, rather than muscle atonia: optogenetic activation of vM GABAergic neurons induces and prolongs REM sleep and chemogenetics inhibition of vM GABAergic neurons reduces REM sleep quantities. Another recent study specifically targeted the vmM (GiV, GiA and RMg) to demonstrate that genetic inactivation of vmM GABAergic/glycinergic neurons does not affect sleep architecture including REM sleep, but suppresses muscle atonia (Valencia Garcia et al., 2018). Thus, if the optogenetic/chemogenetic study (Weber et al., 2015) primarily targeted the LPGi, rather than the vmM, it is probable that vmM and LPGi inhibitory neurons may play distinct roles in muscle atonia and REM sleep, respectively. Further investigation to reconcile these observations is required.

#### 2.1.4 Pedunculopontine tegmental nucleus and laterodorsal tegmental nucleus

The pedunculopontine tegmental nucleus (PPT) and laterodorsal tegmental nucleus (LTD) are a brainstem cholinergic system and have long been implicated in REM sleep, arousal and cortical desynchronization (McCarley, 2007; Mena-Segovia and Bolam, 2017; Scammell et al., 2017; Weber and Dan, 2016). Although pontine cholinergic neurons were originally thought to play a causal role in REM sleep induction, the exact role of the PPT/LDT in REM sleep remains to be determined fully (Grace and Horner, 2015; Grace, 2015; Kroeger et al., 2017; Van Dort et al., 2015).

***Anatomy.*** The PPT and LDT are located within the caudal cholinergic column (Ch5 and Ch6) and contain a heterogeneous population of cholinergic, glutamatergic and GABAergic neurons (Clements and Grant, 1990; Wang and Morales, 2009; Ford et al., 1995). Subpopulations of these cell classes expressed calcium-binding proteins (calbindin, calretinin, and parvalbumin) and they are heterogeneously distributed within the PPT (Martinez-Gonzalez et al., 2012).

The PPT/LDT receives inputs from diverse areas of the brain, including the cortex, thalamus, hypothalamus, pons, cerebellum, medulla, spinal cord and the basal ganglia (Semba and Fibiger, 1992; Saper and Loewy, 1982; Martinez-Gonzalez et al., 2011). Within the brainstem, DRN and LC neurons project to the PPT (Jones and Yang, 1985; Vertes, 1991), with preferential projection from DRN serotonergic neurons to non-cholinergic neurons (Steininger et al., 1997).

Both cholinergic and non-cholinergic axonal projections have been traced at the single cell resolution (Martinez-Gonzalez et al., 2011; Mena-Segovia and Bolam, 2017; Mena-Segovia et al., 2008). Outputs include, but are not limited to the thalamus, basal ganglia, BF, hypothalamus, LC, pontine reticular formation, ventral tegmental area (VTA), SLD and DRN (Cornwall et al., 1990; Ford et al., 1995; Luppi et al., 1995; Martinez-Gonzalez et al., 2011; Mena-Segovia and Bolam, 2017; Semba et al., 1990). Interestingly, the topographical organization of ascending cholinergic innervation was also described (Mena-Segovia and Bolam, 2017): rostral PPT cholinergic neurons preferentially innervate motor-related areas, such as the basal ganglia, whereas LDT cholinergic neurons preferentially innervate limbic-related areas, such as the VTA.

***Activity.*** Different cell types show distinct state-dependent firing patterns: cholinergic neurons are most active during wakefulness and REM sleep, whereas glutamatergic and GABAergic neurons appear to be maximally active either during wake, REM sleep or during both wake and REM (Datta and Siwek, 2002; Steriade et al., 1990; el Mansari et al., 1989; Cox et al., 2016; Boucetta et al., 2014; McCarley and Hobson, 1971). Consistent with these findings, Fos mapping studies have also shown that both cholinergic and GABAergic neurons are active during REM sleep in both cats and rats (Verret et al., 2005; Maloney et al., 1999; Torterolo et al., 2001).

***Function.*** There is an ongoing debate on the functional role of the PPT/LDT in the initiation and maintenance of REM sleep. Lesion (Petrovic et al., 2013; Shouse and Siegel, 1992; Webster and Jones, 1988; Sastre et al., 1981) and pharmacological (Boissard et al., 2002; Gnadt and Pegram, 1986; Deurveilher et al., 1997; George et al., 1964; Amatruda et al., 1975; Baghdoyan et al., 1984; Coleman et al., 2004; Pollock and Mistlberger, 2005) studies have provided inconsistent and contradictory results. Recently, chemogenetic activation of PPT neurons was performed (Kroeger et al., 2017): glutamatergic activation increases the duration of wakefulness, while GABAergic activation moderately reduces the duration of REM sleep. Consistent with pharmacological studies (Boissard et al., 2002; Grace et al., 2014), chemogenetic activation of cholinergic neurons has no effect on REM sleep, but promotes light NREM sleep. On the other hand, electrical stimulation of LDT neurons increases the number of REM sleep bouts (Thakkar et al., 1996). Similarly, optogenetic activation of PPT/LDT cholinergic neurons during NREM sleep promotes REM sleep (Van Dort et al., 2015). While the current consensus is that PPT/LDT neurons play a modulatory role in REM sleep generation (Grace and Horner, 2015), state-dependent coordination of PPT/LDT neuronal firing and its influence on downstream nuclei (e.g., SLD and vlPAG) and REM sleep induction need to be fully explored.

#### 2.1.5 Locus coeruleus

Norepinephrine (NE)-producing neurons are located across brainstem nuclei with diverse populations projecting to numerous regions. Of particular interest is the LC, which is one of the most intensively investigated brainstem nuclei in terms of developmental origin, molecular profiles, anatomical connectivity, and physiological and pathophysiological functions (Aston-Jones and Cohen, 2005; Robertson et al., 2013; Schwarz and Luo, 2015).

***Anatomy.*** The LC is located in the dorsal region of the caudal pons, lateral to the LDT and dorsal to the caudal part of the SLD in mice. All LC neurons produce NE by converting from DA using dopamine-beta-hydroxylase (Dbh). Neuropeptide galanin is also expressed in the majority (up to 80%) of LC neurons (Holets et al., 1988; Robertson et al., 2013). Two morphological classes of LC neurons, multipolar and fusiform cells, have been described in the rat (Grzanna and Molliver, 1980; Swanson, 1976). Thus, although all LC neurons are NE-producing, they are not necessarily homogeneous.

LC neurons receive inputs from other arousal systems and project widely throughout the CNS (Kebschull et al., 2016; Luppi et al., 1995; Schwarz and Luo, 2015; Schwarz et al., 2015). Recently, the anatomical input-output relationship of LC-NE neurons was comprehensively characterized by using advanced viral tracing approaches (Schwarz et al., 2015). LC-NE neurons projecting to diverse brain regions receive inputs from similar areas. Thus, they integrate information from, and broadcasts to, many brain regions. However, there is also specificity. For example, medulla-projecting LC-NE neurons receive disproportionally smaller input from the central amygdala than other LC-NE neurons. Although LC neurons were previously thought to be the only source of NE projections to the cortex, NE neurons from other brainstem nuclei also project to the cortex (Robertson et al., 2013). In the context of REM sleep regulation, LC-NE neurons receive strong GABAergic inputs from the vlPAG, and both the dorsal and lateral paragigantocellular nuclei (DPGi and LPGi), which contain REM-on neurons (Gervasoni et al., 2000; Luppi et al., 2017; Verret et al., 2006).

***Activity and function***. LC neurons are generally REM-off (Aghajanian and VanderMaelen, 1982; Aston-Jones and Bloom, 1981; Hobson et al., 1975; McGinty and Harper, 1976). Thus, they promote arousal and play an antagonistic role in REM sleep. While LC-NE activity correlates with pupil diameter during wakefulness (Aston-Jones and Cohen, 2005), LC-NE neurons are virtually silent during REM sleep. Indeed, the pupil size during REM sleep is minimum (Yuzgec et al., 2018).

NE inhibits PPT/LDT cholinergic neurons (Luebke et al., 1992; Williams and Reiner, 1993) and pharmacological enhancement of NE transmission suppresses REM sleep (Gervasoni et al., 2002; Jones, 1991b; Jones et al., 1969). However, optogenetic inhibition of LC neurons did not alter REM sleep (Carter et al., 2010). These conflicting observations may be reconciled in the future by characterizing the remaining NE containing brainstem nuclei (i.e., A1, A2, A5, A7, LC and subcoeruleus). Indeed, a moderate number of NE neurons (A1 and A2) displayed Fos expression after the recovery of REM deprivation, suggesting that non-LC NE neurons may be important for REM sleep regulation (Leger et al., 2009). Thus, in addition to detailed anatomical study across these nuclei, the exact role of brainstem NE neurons in REM sleep regulation still need to be fully characterized.

#### 2.1.6 Dorsal raphe nucleus

Serotonergic (5-HT) neurons can be found in the raphe nuclei, of which the dorsal raphe nucleus (DRN) (B5 cell group) is the largest serotonergic nucleus (Dahlstrom and Fuxe, 1964; Jacobs and Azmitia, 1992). The role of serotonin in sleep was originally proposed by Jouvet (Jouvet, 1972). However, it has since been shown that DRN serotonergic neurons are wake-promoting and suppress REM sleep, similar to LC-NE neurons (McGinty and Harper, 1976; Trulson and Jacobs, 1979). Nevertheless, because of the diversity of cell types within the DRN as well as serotonin receptors across brain regions, the role of serotonin in sleep remains elusive.

***Anatomy.*** The DRN is located in the midline of the brainstem, ventral to the cerebral aqueduct, occupying the ventral part of the PAG. The DRN neurons are composed of serotonergic, glutamatergic and GABAergic neurons, many of which also express a variety of neuropeptides, such as galanin and substance P (Monti, 2010b). A subset of serotonergic neurons also co-release glutamate (Fischer et al., 2014). There is a small population of DA neurons in the DRN (Cho et al., 2017; Dougalis et al., 2012; Hokfelt et al., 1984).

The DRN receives GABAergic inputs from multiple regions, such as the BF, hypothalamus, substantia nigra, VTA, ventral PAG, rostral pontine reticular nucleus, and dorsal gigantocellular nucleus (Gervasoni et al., 2000; Luppi et al., 2008). DRN neurons also receive inputs from neurons releasing a variety of neurotransmitters and neuropeptides, such as histamine, DA, NE, ACh, orexin/hypocretin and melanin-concentrating hormone (MCH) (Panula et al., 1988; Sakai et al., 1977; Beckstead et al., 1979; Saavedra et al., 1976; Woolf and Butcher, 1989; Lee et al., 2005a; Hervieu et al., 2000; Kilduff and de Lecea, 2001).

DRN neurons innervate a wide range of areas, including the cerebral cortex, amygdala, BF, thalamus, preoptic and hypothalamic areas, LC, and pontine reticular formation (Imai et al., 1986; Peyron et al., 1998a). Based on the distribution of cell types and anatomical projections, six subdivisions of the DRN have been proposed in rats (Lowry et al., 2008; Monti, 2010b).

***Activity.*** Although the majority of 5-HT neurons are active during wake and virtually inactive during REM sleep (McGinty and Harper, 1976; Trulson and Jacobs, 1979), heterogeneity of their firing has been reported in cats, rats and mice (Allers and Sharp, 2003; Hajos et al., 1995; Sakai and Crochet, 2001; Urbain et al., 2006; Sakai, 2011). For example, a fourth of the DRN neurons are sleep-active and around one-fifth are active during both wakefulness and REM sleep in mice. They are topographically organized (Sakai, 2011). DRN-DA neurons were observed to be most active during wakefulness (Cho et al., 2017).

***Function.*** Pharmacological studies indicate that GABAergic inputs to the DRN play a key role in REM induction (Gervasoni et al., 2000; Nitz and Siegel, 1997). GABA concentrations in the DRN increase during REM sleep and pharmacological activation/inactivation of GABAa receptors increases and decreases REM sleep, respectively. GABAergic neurons in the vlPAG, DPGi and LPGi seem to provide the source of these GABAergic inputs.

While DRN DA neurons can induce arousal (Cho et al., 2017), the serotonergic effects on REM sleep are complex, depending on the expression and location of receptor subtypes (Monti, 2010a): for example, 5-HT1A receptor expressing DRN 5-HT neurons inhibit adenylate cyclase. The administration of 5-HT1A agonists into the DRN reduces 5-HT concentration and enhances REM sleep (Portas et al., 1996). On the other hand, 5-HT1A receptor also expresses in downstream PPT/LDT cholinergic neurons. Microinjection of 5-HT1A receptor agonists into the LDT suppresses REM sleep (Monti and Jantos, 2004). Thus, serotonergic effects on REM sleep are site-specific.

In summary, although it is widely thought that most serotonergic neurons are wake-promoting and REM-sleep-inhibiting, the exact roles of DRN neurons in sleep are still elusive. Because serotonin and sleep are closely related with depression, further studies on DRN neurons would be relevant from both basic scientific and clinical viewpoints.

### 2.2 Hypothalamic REM sleep-regulating circuits

The hypothalamus consists of highly heterogeneous cell populations (Romanov et al., 2017) and contributes to diverse biological functions including sleep related functions, such as the circadian rhythm, the stabilization of the sleep-wake cycle, and REM sleep regulation. In particular, a wide range of neuropeptides play a role in sleep regulation (Steiger and Holsboer, 1997). Recent genetic-based circuit studies have significantly contributed to the advancement in this topic. In this section, we summarize the 1) molecular features, 2) anatomical features, 3) neural activity during REM sleep, and 4) gain- and loss-of-function studies, of three neuropeptidergic systems within the hypothalamus (Fig. 2): orexin/hypocretin, melanin-concentrating hormone (MCH), and galanin.

#### 2.2.1 Orexin/hypocretin

Hypothalamic orexin/hypocretin (OH) neurons heavily innervate REM sleep-suppressing brainstem regions, including the LC, DRN, and vlPAG. A deficit in this system results in narcolepsy, with pathological intrusion of REM sleep, called cataplexy. Thus, this system is crucial for the physiological regulation of REM sleep by stabilizing wakefulness.

***Molecular features.*** OH is a neuropeptide, consisting of orexin A (hypocretin 1) and orexin B (hypocretin 2) derived from a common precursor peptide, prepro-orexin (de Lecea et al., 1998; Sakurai et al., 1998). OH can activate two G-protein-coupled receptors, OX1R and OX2R (Sakurai et al., 1998). The former has greater affinity for orexin A than orexin B whereas the latter has similar affinity for both types. OX1R is coupled to the Gq/11 to activate phospholipase C whereas OX2R is coupled to both Gq/11 and Gi.

***Anatomy.*** OH neurons are exclusively located in the lateral hypothalamus (LH) and posterior hypothalamus (PH). They coexpress dynorphin, galanin, prolactin, neuronal activity-regulated pentraxin and glutamate (Chou et al., 2001; Risold et al., 1999; Abrahamson et al., 2001; Tsujino and Sakurai, 2009). Many OH neurons also express vesicular glutamate transporters, but not GAD67, suggesting that they are also glutamatergic (Rosin et al., 2003).

OH neurons receive inputs from the lateral parabrachial nucleus, VLPO, medial and lateral preoptic areas, BF, posterior/dorsomedial hypothalamus, VTA, and median raphe nuclei (Sakurai et al., 2005; Yoshida et al., 2006). OH neurons also receive inputs from Lhx6-positive GABAergic neurons in the zona incerta (ZI) (Liu et al., 2017).

OH neurons widely project to various regions (Peyron et al., 1998b): in addition to innervating areas within the hypothalamus, the densest projection can be found in the LC. OH neurons also project to the septal nuclei, BNST, the paraventricular and reuniens nuclei of the thalamus, ZI, subthalamic nucleus, PGA (including vlPAG), substantia nigra, DRN, parabrachial area, PPT/LDT, medullary reticular formation, inferior colliculus, and the nucleus of the solitary tract. The expression pattern of OXRs is generally consistent with the innervation pattern of OH neurons. The distribution of OX1R and OX2R is partially overlapped, implying distinct functional roles (Trivedi et al., 1998; Marcus et al., 2001; Lu et al., 2000b).

***Activity.*** OH neurons are generally REM-off. They discharge during active wakefulness and decrease their firing during quiet wakefulness, but still respond to sensory stimulation. They are silent during sleep including both NREM and REM sleep (Hassani et al., 2009; Lee et al., 2005b; Mileykovskiy et al., 2005).

***Function.*** Although OH neurons receive inputs from various wake-promoting neurons, their effects on OH neuronal activity are complex. For example, carbachol and histamine have excitatory effects on OH neurons (Brown et al., 2002; Sakurai et al., 2005) whereas serotonergic neurons have inhibitory effects (Muraki et al., 2004; Sakurai et al., 2005). Effects of noradrenergic neurons are mixed (Carter et al., 2012; Yamanaka et al., 2006; Hara et al., 2001). OH neurons are inhibited by a subset of GABAergic neurons (Lhx6+) in the ventral ZI to induce NREM sleep (Liu et al., 2017).

OH activates monoaminergic systems, including LC-NE, VTA-DA, DRN-5-HT, and histaminergic cells (Yamanaka et al., 2002; Brown et al., 2002; Hagan et al., 1999; Nakamura et al., 2000), consistent with the notion that OH plays a causal role in arousal (Adamantidis et al., 2007). However, the effects of OH on cholinergic neurons depend on the cholinergic nucleus. For instance, orexin A injected into the LDT increases the time spent in wakefulness and decreases the time spent in REM sleep (Xi et al., 2001). Orexin A induces excitation of cholinergic neurons in the LDT (Takahashi et al., 2002) and BF (Eggermann et al., 2001). On the other hand, orexin A can indirectly inhibit PPT cholinergic neurons by activating PPT GABAergic interneurons as well as GABAergic neurons in the substantia nigra pars reticulata (Takakusaki et al., 2005).

Effects of OH neuron activation on MCH neurons are generally inhibitory, but can also be excitatory (Apergis-Schoute et al., 2015; van den Pol et al., 2004; Hassani et al., 2009). The inhibitory effect of OH cells on MCH neurons is probably due to the recruitment of local GABAergic neurons via OH activation, but not glutamatergic or dynorphinergic effects (Apergis-Schoute et al., 2015).

In summary, findings are generally consistent with the notion that OH neurons play a role in the stabilization of wakefulness by interacting with other wake/sleep-promoting neurons. However, detailed synaptic and circuit mechanisms remain to be fully characterized. In addition, although the OH system plays a causal role in narcolepsy (Pintwala and Peever, 2017; Sakurai, 2007; Thannickal et al., 2000; Peyron et al., 2000; Lin et al., 1999; Chemelli et al., 1999; Tsujino and Sakurai, 2009), the cause of narcolepsy is still unclear.

#### 2.2.2 Melanin-concentrating hormone

While OH neurons stabilize wakefulness, melanin-concentrating hormone (MCH) neurons have the opposite effect on the regulation of sleep-wake states by increasing REM and NREM sleep.

***Molecular features.*** MCH was first discovered in fish (Kawauchi et al., 1983; Rance and Baker, 1979) and later in the mammalian brain (Vaughan et al., 1989; Nahon et al., 1989). MCH is produced from a preproprotein, called prepro-MCH, which also encodes neuropeptide-glutamic acid-isoleucine (NEI) and neuropeptide-glycine-glutamic acid (NGE). MCH is a 19-amino acid neuropeptide and binds to G-protein-coupled receptors, termed MCHR1 (or GPR24) and MCHR2. As MCHR1 is coupled with Gai/o and Gaq proteins, MCHR1 activation causes a strong inhibition of neurons (Hawes et al., 2000). MCHR2 gene shows species differences: in rodents (the rat, mouse, hamster, and guinea pig) and rabbits, MCHR2 is a pseudogene, thus non-functional whereas in carnivores (the dog and ferret) and primate (rhesus macaque and human), MCHR2 is functional and expressed in the brain (such as the claustrum) (Tan et al., 2002). MCHR2 is coupled to Gq proteins, which trigger intraceullular signaling. However, the function of MCHR2 remains unknown.

***Anatomy.*** MCH neurons are primarily located in the LH and ZI. The distribution of MCH neurons shows sexually dimorphic patterns in rats. For example, MCH neurons can be found in the LDT of female but not male rats (Rondini et al., 2007). Some MCH neurons co-express GAD67 and others express vGluT1 (Jego et al., 2013; Harthoorn et al., 2005).

Anatomical inputs to MCH neurons has been characterized comprehensively (Gonzalez et al., 2016). Of numerous brain regions, the following areas provide strong innervation to MCH neurons: the tuberal nucleus and the periventricular, the lateral, ventromedial and dorsomedial hypothalamic nuclei within the hypothalamus; the nucleus accumbens, BNST, and BF within the cerebral nuclei; and the midbrain reticular nucleus, PAG and VTA within the midbrain.

MCH neurons project to areas throughout the brain. In particular, they heavily innervate the LH, medial septum, medial diagonal band, lateral part of the medial mammillary nucleus, and PPT (Bittencourt et al., 1992).

***Activity.*** A Fos mapping study demonstrated that MCH neurons are strongly active during REM sleep (Verret et al., 2003). This was subsequently confirmed with electrophysiology, whereby MCH neurons were observed to fire exclusively during REM sleep in rats (Hassani et al., 2009). Thus, contrary to OH neurons, MCH neurons are REM-on.

***Function.*** Consistent with these observations, intracerebroventricular injection of MCH increases both NREM and REM sleep in a dose dependent manner (Verret et al., 2003). Similarly, when administered into the DRN, vlPAG and LC, MCH also increases the time spend in REM sleep, with a moderate increase in NREM when injected into the DRN (Lagos et al., 2009; Fujimoto et al., 2017; Monti et al., 2015). Furthermore, MCHR1 antagonist administration decreases REM and NREM sleep (Ahnaou et al., 2008). However, an increase in REM sleep episodes was observed in MCHR1 knockout mice, suggesting that compensatory mechanisms are at play (Adamantidis et al., 2008).

A series of optogenetic studies have shed new light on the function of MCH neurons (Konadhode et al., 2013; Tsunematsu et al., 2014; Jego et al., 2013). Although detailed approaches and optical stimulation protocols varied across reports, all three independent studies confirmed that the activation of MCH neurons increases the time spent in REM sleep. A chemogenetic study also confirmed a similar effect (Vetrivelan et al., 2016). On the other hand, varied optogenetic experimental parameters have also resulted in inconsistent effects being observed. For example, Jego and colleagues only observed increases in REM sleep duration with 20 Hz optical stimulation at the onset of REM sleep, but not with 1 Hz stimulation. Another recent study showed that optogenetic activation of MCH neurons increases both REM and NREM sleep at night (active period), but only increases REM sleep during the light period (inactive period) (Blanco-Centurion et al., 2016).

Optogenetic silencing of MCH neurons did not affect sleep architecture (Tsunematsu et al., 2014; Jego et al., 2013). Although genetic ablation of MCH neurons also had no effect on the total duration of REM sleep, the chronic deletion of MCH neurons altered the diurnal rhythm (Tsunematsu et al., 2014; Vetrivelan et al., 2016). Overall, MCH neurons play a regulatory role in REM sleep and probably NREM sleep too. However, given the activity pattern of MCH neurons across the sleep-wake cycle (∼ 1 Hz, not 20 Hz, during REM sleep), it is still unclear how MCH neurons modulate REM sleep-regulating circuits.

#### 2.2.3 Galanin

Galanin (GAL) is a neuropeptide expressed widely in the brain and peripheral tissue. While GAL has been implicated in the regulation of numerous physiological functions, GAL-positive (GAL+) neurons in the hypothalamus have been found to play a role in sleep regulation. However, the exact function of GAL + neurons in sleep remains uncertain. Here, we summarize the basic molecular and anatomical features of GAL and GAL receptors, and then focus on the function of hypothalamic GAL + neurons in REM sleep regulation.

***Molecular features.*** GAL consists of 29 amino acids (Tatemoto et al., 1983) (30 amino acids in humans). GAL is produced from a 123-animo acid precursor, which is encoded by the *GAL* gene (Crawley, 1995). Three G-protein-coupled GAL receptors have been identified: GALR1 and GALR3 inhibit adenylyl cyclase whereas GALR2 stimulates phospholipase C and increases intracellular inositol triphosphate turnover (Lang et al., 2007; Branchek et al., 2000; Smith et al., 1998; Fathi et al., 1997; Habert-Ortoli et al., 1994).

***Anatomy.*** GAL is widely expressed in the central nervous system, with strong expression in the hypothalamus, medulla, and spinal cord (Merchenthaler et al., 1993; Skofitsch and Jacobowitz, 1986; Melander et al., 1986). In the telencephalon, GAL + neurons can be found in the BNST, BF, and central nucleus of amygdala. In the pons, the DRN and LC also contain a large number of GAL + neurons. GALRs are also widely expressed, but show distinct expression patterns (Mennicken et al., 2002; Kolakowski et al., 1998; O’donnell et al., 1999; Parker et al., 1995), suggesting diverse functions of GAL.

Hypothalamic GAL + neurons are primarily GABAergic, but a recent study identified distinct sub-populations based on single-cell RNA sequencing analysis (Romanov et al., 2017). Although beyond the scope of this review, a subset of cholinergic neurons in the BF also expresses GAL, implicating a role in arousal and memory (Miller et al., 1998; Melander et al., 1985).

***Activity and function.*** Although GAL administration does not promote REM sleep (Toppila et al., 1995), GAL + neurons play a role in REM sleep regulation. Neurons extending dorsally and medially from the VLPO (called the extended VLPO, eVLPO) have been implicated in REM sleep regulation (Lu et al., 2000a; Koyama and Hayaishi, 1994; Szymusiak et al., 1998; Lu et al., 2002). Saper and his colleagues demonstrated that REM sleep duration is correlated with the number of Fos + neurons in the eVLPO - a majority of which are GAL-positive (Lu et al., 2002). eVLPO neurons project to both REM-on/-off regions, including the vlPAG, LDT, DRN and LC (Lu et al., 2006b, 2002). GAL inhibits LC neurons (Seutin et al., 1989), suggesting REM sleep-promoting effects.

GAL + neurons in the dorsomedial hypothalamus (DMH) also regulate REM sleep (Chen et al., 2018). Interestingly, DMH GAL + neurons projecting to the preoptic area are REM-off and optogenetic activation of these neurons suppress REM sleep. The preoptic area also contains MCH neurons. On the other hand, DMH GAL + neurons projecting to the RPA in the vM are REM-on and optogenetic activation of these neurons promote REM sleep. Characterizing anatomical and functional interactions between two distinct GAL + neurons within the DMH would be interesting.

Thus, GAL + neurons can promote and inhibit REM sleep depending on implemented circuits. Further comprehensive anatomical and functional characterization will clarify the role of GAL + neurons in REM sleep. In addition, because GAL expression itself can be changed by responding to anatomical lesions or REM sleep deprivation (Toppila et al., 1995; Cortes et al., 1990), the dynamic regulatory mechanism of GAL expression is also an important issue.

## 3 Computational models of REM sleep

**Fig. 3:**
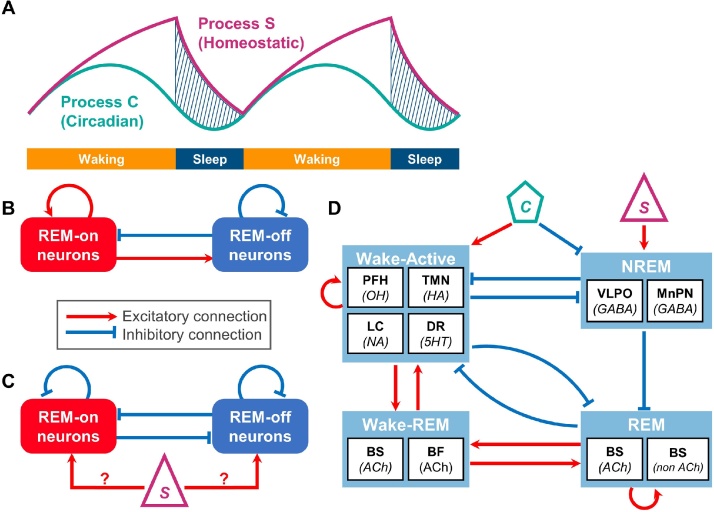
**Computational models of sleep-wake cycles and REM sleep.** (**A**) Two-process model (modified from Borbély 1982). Sleep is regulated by a homeostatic mechanism (Process S) and a circadian mechanism (Process C). Total sleep propensity is represented by the difference between the Processes S and C. (**B**) An elementary component of a Reciprocal Interaction (RI) model. (**C**) An elementary component of a Mutual Inhibition (MI) model. A homeostatic/circadian drive contribute to shifting states. (**D**) An example of an integrative model (modified from Tamakawa et al., 2006). BS, brainstem; BF, basal forebrain; DR, dorsal raphe nucleus; LC, locus coeruleus; MnPN, median preoptic nucleus; PFH, perifornical hypothalamus; TMN, tuberomammillary nucleus; VLPO, ventrolateral preoptic area; 5 H T, serotonin; Ach, acetylcholine; GABA, gamma aminobutyric acid; HA, histamine; NA, noradrenaline; OH, orexin/hypocretin.

The wake-sleep cycle is regulated by complex interactions between at least three fundamental processes: a homeostatic process, a circadian process, and an ultradian process. Computational models of sleep-wake cycles typically incorporate at least one of these processes. The main purpose of these computational models can be (1) to replicate the dynamics of sleep-wake cycles with and without perturbations, such as sleep deprivation and pharmacological manipulations, (2) to replicate the dynamics of neural population activity in sleep/wake regulating circuits, and (3) to explain the relationship between specific neural systems and disease states, such as the relationship between OH neurons and narcolepsy. The level of implementation varies across models, from the more conceptual model to the more detailed neural network models with varied architectures and formalizations. In this section, we provide a brief overview of the computational models of the sleep-wake cycle, with an emphasis on REM sleep. We begin by introducing Borbely’s two-process model (Fig. 3A) to provide a broad context of this topic. We will then focus on computational models of REM sleep regulation which include two major categories of models - the reciprocal interaction (RI) model and the mutual inhibition (MI) model (also known as the flip-flop switch model) (Figs. 3**B and C**). Finally, we briefly summarize integrative models which contain various brain structures. Readers may also refer to other reviews (Weber, 2017; Borbely and Achermann, 1992; Booth and Diniz Behn, 2014).

### 3.1 Two-process model

Borbely’s two-process model offers a conceptual framework of sleep-wake cycles (Borbely, 1982). In this model, a homeostatic sleep-dependent process (Process S) and a circadian process (Process C) play a dominant role in sleep regulation (Fig. 3A). Process S reflects the global changes in cortical slow wave activity (SWA) as a biomarker. Process C can be explained by the activity of the suprachiasmatic nucleus (SCN) (Welsh et al., 2010). Total sleep propensity is represented by the difference between the Processes S and C. After quantitative implementation of this concept (Daan et al., 1984), the two-process model was further extended with an external function to trigger REM sleep (Achermann and Borbely, 1990). Computational models over the last decade have implemented this model with detailed network architectures to reflect experimental observations. For example, Phillips and Robinson have implemented the two-process model in several neural network models which uses different neural population firing rate formalisms (Robinson et al., 2011; Phillips and Robinson, 2008, 2007). In the following sections, we focus on computational models of REM sleep regulation.

### 3.2 Reciprocal interaction model

The RI model consists of an excitatory REM-on population interacting with an inhibitory REM-off population (Fig. 3B). The first of its kind was produced by McCarley and Hobson with REM-on neurons in the pontine cholinergic field interacting with REM-off neurons in the LC (McCarley and Hobson, 1975). A predator-prey interaction model with Lotka-Volterra equations was utilized in this model, whereby the activity of REM-off neurons (predator) gradually decays due to self-inhibition, which results in increased activity of REM-on neurons (prey). Although this model mimicked the time course of neural firing of REM-on and REM-off neurons, it was too sensitive to perturbations and did not include the circadian rhythm. Subsequently, the original model was extended by introducing a limit cycle as well as circadian variation (McCarley and Massaquoi, 1986). This limit cycle reciprocal interaction model (LCRIM) was applicable to human sleep data as well as for a simulation of pharmacological experiments. An integrated model was further developed to generate qualitatively realistic sleep-wave cycles by linking the two-process model with the LCRIM (Massaquoi and McCarley, 1992).

Recent models incorporated the dynamics of the RI model into a more physiologically based model (Booth et al., 2017; Behn and Booth, 2012; Diniz Behn and Booth, 2010; Behn et al., 2007; Tamakawa et al., 2006), which we summarize in the section below. Although the original model was inspired by excitatory and inhibitory interactions between cholinergic and monoaminergic systems, experimental evidence (discussed above) over the last decade suggests that this reciprocal interaction may not be sufficient for REM sleep generation.

### 3.3 Mutual inhibition model

The MI model (or flip-flop switch model) consists of mutual inhibitory interactions between REM-on and REM-off neurons (Fig. 3C). The original model was conceptually proposed for wake-sleep regulation where sleep-promoting VLPO GABAergic neurons and wake-promoting monoaminergic neurons mutually inhibit each other. The wake stabilizing effects of OH neurons were also represented as a ‘finger’ of the flip-flop switch (Saper et al., 2001). Later, Lu and his colleagues extended this concept to REM-NREM regulation by experimentally demonstrating inhibitory REM-on and REM-off neurons within the brainstem (Lu et al., 2006b). Since then, several computational models incorporated this mutual inhibition into their network architecture (Grace et al., 2014; Dunmyre et al., 2014; Kumar et al., 2012; Rempe et al., 2010; Behn and Booth, 2012).

In this model, mutually inhibiting components provide a bistable feedback loop. Because a key feature of this circuit is self-reinforcing to stabilize a particular state, additional inputs to inhibitory components play a key role in shifting the balance of mutual inhibition, that is, state change. Such inputs can reflect circadian and/or homeostatic drives. However, the neural basis of such drives remains unknown.

Of recent models, Booth and her colleagues developed a simple, but elegant dynamical system model to implement this MI model with their neural population firing rate and neurotransmitter formalism (Dunmyre et al., 2014). By coupling two flip-flop switches together, the model could capture rat sleep behavior including recovery after REM sleep deprivation.

Although most models have been developed as a population firing rate model with varied formalisms, a network model with leaky integrate-and-fire model neurons was also developed in order to assess the effect of muscarinic receptor anatagonism on REM-on subcoeruleus activity (Grace et al., 2014). In this model, MI between REM-on and –off neurons within the vlPAG was implemented with a ramping input to REM-off neurons. A similar network model with the large number of model neurons needs to be developed to fully capture the dynamics of sleep-wake cycles, not just the transition from NREM to REM sleep.

### 3.4 RI model versus MI model

What is the similarity and difference between the RI and MI models? How do each of these network motifs respond to perturbations? Which motif plays a primary causal role for REM sleep transitions? Although answers to these questions remain unclear, these issues were addressed by Diniz Behn and her colleagues (Behn et al., 2013). By using minimal RI and MI models with varied implementations of homeostatic drive, they identified conditions for the generation of REM-NREM sleep cycles and investigated the robustness of REM-NREM sleep cycles by analyzing the response of model dynamics to manipulation of synaptic interactions and self-modulatory inputs.

The RI model results in stable limit cycle oscillations rather than a single fixed point, meaning that continuous state shifts are inherited in this system. On the other hand, the MI model stabilizes the brain state. In contrast to the RI model, extrinsic inputs or parameter change are required to change the state within the MI model. Thus, the homeostatic drive plays a crucial role in REM-NREM sleep cycles in the MI model. These results suggest that the neuronal populations associated with the causal REM sleep network may be identified by evaluating distinct responses in REM sleep dynamics to experimental modulation of specific network components. The combination of experimental approaches with these types of computational studies will be helpful to interpret the dynamics of REM-on and REM-off neural firing as well as the effect of perturbations (e.g., optogenetics) on REM-NREM sleep cycles.

### 3.5 Integrative models

While computational models for REM sleep can be conceptually categorized into two major categories, researchers have also developed integrative models that include various brain regions in accordance with experimental observations. For example, Tamakawa et al (2006) developed an ambitious integrative model consisting of 10 subcortical nuclei across the BF, hypothalamus and brainstem, which can be categorized into four functional units: sleep-active, wake-active, REM-active (REM-on), and wake-REM-active groups (Fig. 3D). Despite the large number of parameters, this ‘quartet’ network successfully reproduced the dynamics of neural firing in each component across sleep-wake cycles (Tamakawa et al., 2006). Subsequently, different neural network models have been developed with varied network architectures and mathematical formalisms (Grace et al., 2014; Kumar et al., 2012; Rempe et al., 2010; Behn and Booth, 2012; Diniz Behn and Booth, 2010; Behn et al., 2007; Booth and Diniz Behn, 2014). As new experimental discoveries are made, such integrative models should be also updated. To date, at least two approaches are missing in this field: first, there are few computational models that implement the effects of optogenetic stimulation on REM sleep regulation (Carter et al., 2012). Second, a large-scale realistic network model with spiking model neurons has not been developed to the best of our knowledge.

## 4 Conclusion and future directions

In summary, REM sleep-regulating circuits are widely distributed across the brainstem and hypothalamus, utilizing diverse neurotransmitters and neuropeptides. Thus, REM sleep-regulating circuits are a highly robust and complex system. While experimental findings are still fragmented and mostly qualitative, it is crucial to understand this complexity quantitatively by incorporating advanced technologies with computational modeling. In particular, computational models reflecting the latest experimental evidence are urgently required, together with quantifying anatomical/synaptic connections as well as characterizing the dynamics of neural ensembles in the hypothalamus and brainstem in a cell type-specific fashion.

To better understand REM sleep, at least five key questions remain to be addressed: the first issue is to determine the evolutionary origin of REM sleep. Given the complexity and redundancy of the regulatory circuit, it is not surprising that the primitive elements of REM sleep can be found in lower vertebrates (Shein-Idelson et al., 2016). Although adult mice have been a popular choice as an animal model, further comparative (Joiner, 2016) and developmental studies (Hayashi et al., 2015; Robertson et al., 2013) will help shed new light on the mechanism underlying REM sleep regulation. Advanced anatomical methods, lineage tracing, and genome engineering may play an important role to this end.

The second issue is to dissect the anatomical complexity of REM sleep-regulating circuits at various levels. For example, what neurotransmitters and neuropeptides are utilized? How many cell types are contributing to REM sleep regulation? How are they anatomically connected in a cell-type-specific manner? Advanced genetic and single-cell profiling technologies, as well as the latest anatomical methods should be applied to address these questions (Weber and Dan, 2016). In addition, *in vitro* slice experiments will add quantitative information about synaptic connections.

The third issue is to determine the neural basis of homeostatic control of REM sleep. Although REM sleep deprivation has long been used for REM sleep experiments, the neural mechanisms underlying the homeostatic control of REM sleep remains elusive. A closed-loop experiment with optogenetic manipulations to manipulate the duration of REM sleep may be a promising approach to address this issue without causing stress compared to conventional approaches. This closed-loop approach will also offer an opportunity to explore the functions of REM sleep.

The forth issue is to decipher the dynamics of neural ensembles for REM sleep regulation. Since the identification of REM-on and –off neurons in 1970s, it is still unclear how these functionally distinct cell populations interact with each other. Monitoring neural ensembles across hypothalamic/brainstem areas and incorporating these findings with computational approaches will provide promising insights into the neural dynamics of REM sleep. Although realistic computational models with spiking neurons are also worth developing, quantitative descriptions of the electrophysiology and anatomy are still scarce. Efforts similar to those for cortical circuits are unmet needs in this field.

The final and most fundamental issue is to understand the function of REM sleep. Why did REM sleep emerge only in mammals, birds and some reptiles? Why does the duration of REM sleep decrease as the brain matures? What are the differences in memory-related neural processes between REM and NREM sleep? And why do we dream? Although several influential hypotheses have been proposed during the 1960s to 80s (Crick and Mitchison, 1983; Hobson and McCarley, 1977; Davenne and Adrien, 1984; Roffwarg et al., 1966), they have not been fully tested experimentally. Accumulating evidence suggests the role of REM sleep in memory consolidation (Boyce et al., 2017; Stickgold and Walker, 2013; Poe, 2017; Sara, 2017; Rasch and Born, 2013; Siegel, 2001; Peever and Fuller, 2017). While controversy surrounding this hypothesis has persisted, a recent study used optogenetics in mice to demonstrate that theta rhythm during REM sleep plays a causal role in spatial and contextual memory consolidation (Boyce et al., 2016). Another recent imaging study showed that newly formed synapses of cortical layer 5 pyramidal cells can be selectively eliminated and maintained via dendritic calcium spike-dependent mechanisms during REM sleep (Li et al., 2017). Thus, with the advent of recent revolutionary technologies, it is now an exciting period to revisit early hypotheses for a better understanding of REM sleep.

## Author contributions

CH, AP and SS wrote the manuscript.

## Conflict of interest

The authors declare no conflict of interest.

## Glossary

ACh: acetylcholine
BF: basal forebrain
BNST: bed nucleus of the stria terminalis
DA: dopamine
DMH: dorsomedial hypothalamus
DpMe: deep mesencephalic reticular nucleus
DRN: dorsal raphe nucleus
EEG: electroencephalogram
eVLPO: extended area of the ventrolateral preoptic area
GABA: γ-aminobutyric acid
GiA: alpha gigantocellular nucleus
GiV: ventral gigantocellular nucleus
Glu: glutamate
Gly: glycine
LC: locus coeruleus
LDT: laterodorsal tegmental nucleus
LPB: lateral parabrachial nucleus
LPGi: lateral paragigantocellular nucleus
MCH: melanin-concentrating hormone
MPB: medial parabrachial nucleus
OH: orexin/hypocretin
PAG: periaqueductal gray
PPT: pedunculopontine tegmental nucleus
REM: rapid eye movement
RMg: raphe magnus
RPA: nucleus raphe pallidus
SLD: sublaterodorsal nucleus
vlPAG: ventrolateral periaqueductal gray
VLPO: ventrolateral preoptic area
vM: ventral medulla
vmM: ventromedial medulla
VTA: ventral tegmental area
ZI: zona incerta
